# Supplementary material for: Visual odometry of Rhinecanthus aculeatus depends on the visual density of the environment
Source: Commun Biol. 2022 Oct 1;5:1045. doi: 10.1038/s42003-022-03925-5 (PMC9526725; doi:10.1038/s42003-022-03925-5)
Supplement: Supplementary file 2 — Supplementary Information [file 42003_2022_3925_MOESM2_ESM.pdf]

Supplementary Information: Visual odometry in a freely-swimming fish

C. Karlsson[1], J. K. Willis[1], M. Patel[1], and T. Burt de Perera[1]

[1]Department of Zoology, Research and Administration Building, 11a Mansfield Road, Oxford, OX1 3SZ

Contents

1 Supplementary Methods: additional information **i**

1.1 Controlling for use of external cues i

1.2 Study species and population sampling ii

1.3 Full distance training protocol v

2 Supplementary Results: additional information **vi**

2.1 Distance estimates across visual treatments vi

2.2 Absolute turning position by starting area position across visual treatments vii

2.3 Distance estimate variability xi

2.4 Swimming speed across visual treatments xii

1 Supplementary Methods: additional information

1.1 Controlling for use of external cues

The start area was moved between three different positions within the tunnel to prevent the fish from being able to use positional cues internal or external to the maze. Each session the start area and infrared proximity sensory moved within the tunnel to a new position. These are as follows: +0 - the baseline positon with the rear perforated screen 0.02m from the rear of the tank; +10 - 10cm distal movement from the baseline +0 position; +20 - 20cm distal movement from the baseline +0 position. The rear perforated screen was shifted accordingly to maintain the start area length at 0.3m. This was to account for different individuals starting their distance estimates at different points within the start area. Supplementary Figure 1 is a schematic of these positions.

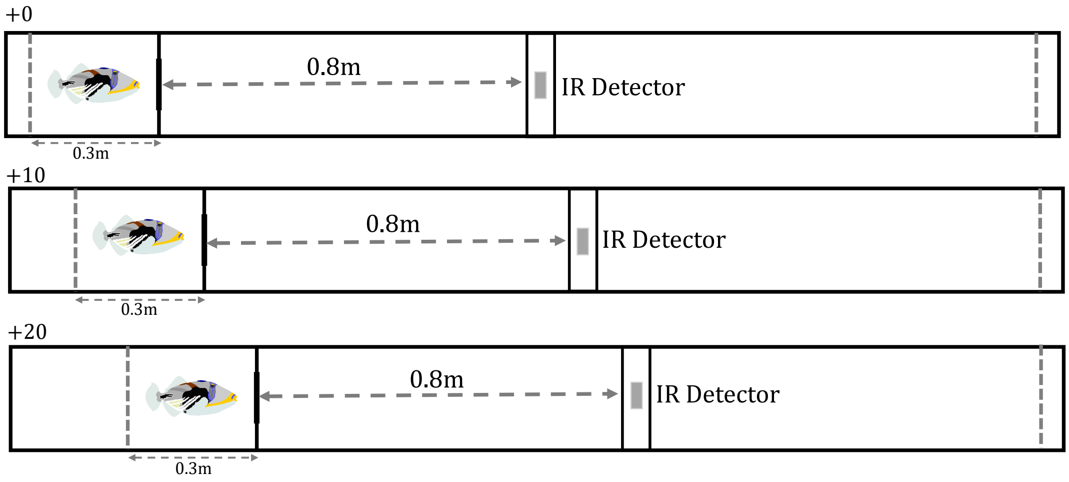

Supplementary Figure 1: Schematic of the three start area positions. Each position is located increasingly distally through the tunnel by 0.10m increments (labelled +0, +10 and +20). The rear perforated white screen moves accordingly to ensure the start area length is kept at 0.30m. The infrared detector also moves accordingly to maintain the target distance of 0.80m.

1.2 Study species and population sampling

The Picasso triggerfish, *Rhinecanthus aculeatus*, was chosen as our study species as it has proven to be trainable in complicated long-term experiments, and responds well to training in operant conditioning paradigms[1,2]. Individuals are also naturally territorial, with males defending discrete territories with female harems. Within these harems, females defend their own territories within the larger male territory. This makes them amenable to individual housing without compromising welfare and altering natural behaviours.

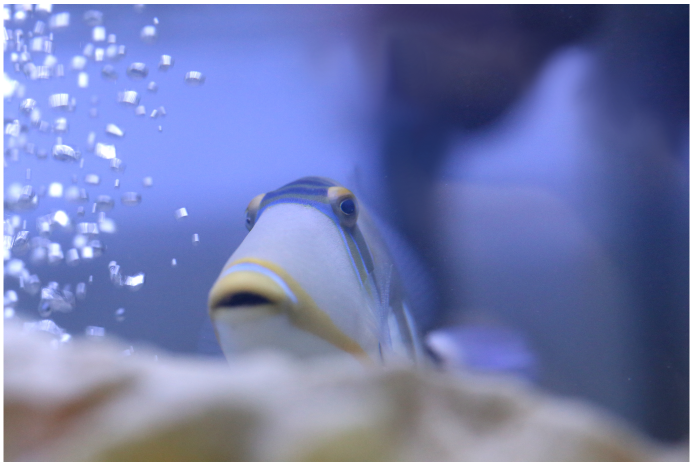

Supplementary Figure 2: An experimental subject used in experiments, hiding behind tank enrichment. Photo taken by J.K. Willis.

Tank design and enrichment was designed following a period of field work filming Picasso triggerfish in their natural environment on reefs off Lizard Island in Australia (work led by Cait Newport, 2018). Individuals were housed in tanks measuring 0.45m width x 0.35m height x 0.75m length with coral gravel, sand, rocks and caves provided for enrichment. All tanks were provided with an individual airline to ensure sufficient aeration, and were inter-connected to a communal sump system ensuring high flow and water cycling. Tanks were cleaned twice weekly, with water parameters monitored weekly (Nitrates at max 15ppm, Nitrite at 0ppm, Ammonia at 0ppm, and salinity at 35ppt). Fish were rewarded with Ocean Nutrition formula one and two pellets and krill pacifica during experiments, and supplemented with a diet of krill, lance fish and cockles at the end

of each training day. Supplement feeds were given as a combination of tweezer feeds and scatter feeds to encourage active foraging.

Power analysis was conducted prior to experiments to estimate required sample size and minimise the number of individuals used to answer the question. Initial sample size was calculated using the 'pwr' package in R (The R project, version 3.6.1). To achieve a power of 80%, with a significance level of 0.05 for a large predicted effect size (set at 0.8, using the Cohen 1988 approximation[3]), with four treatment levels, the required sample size was calculated as 5.3 individuals.

We tested 6 individuals that had previously undergone behavioural training in a distance estimation accuracy task[4]. All had been housed in captivity for under 9 months by experiment completion. All fish were of similar size (10-14cm standard length), but as they were wild-caught we could not determine sex or age. All fish underwent a standard 4-week quarantine procedure prior to inclusion in the main aquarium system to ensure only healthy individuals were used in experiments. Pre-training involved teaching the fish to associate tweezers with food, and all fish were trained to swim voluntarily into a 2l container to transfer them between their home tank and experiment tank without causing stress.

In the main experiment, fish were trained up to testing criterion (80% performance across 3 consecutive sessions), and were subsequently tested in their abilities to reproduce learned distances across four visual treatments, one after the other. Treatments were provided as follows: Test 1 (0.02m stripes), training condition; Test 2 (0.01m stripes); Test 3 (0.02m checkerboard); Test 4 (horizontal stripes). Four fish (ID: A, B, D and E) completed all four testing treatments, but Fish C and F experienced loss of motivation and were unable to continue the distance estimation task following Test 2 and Test 3 respectively. These fish were unable to reliably complete the 5 training trials at 80% accuracy that preceded each block of 5 testing trials. When this continued across over 10 consecutive sessions, fish were removed from experiments. Sample sizes across treatments were therefore as follows: Test 1 - 6 fish, Test 2 - 6 fish, Test 3 - 4 fish, Test 4 - 5 fish. Following

Fish	Test 1	Test 2	Test 3	Test 4
A	43	44	45	45
B	45	45	44	44
C	45	44	0	0
D	45	44	43	44
E	45	43	44	41
F	45	45	0	42

Supplementary Table 1: Number of testing trials meeting criteria for analysis, split by fish across treatments.

video analysis of these testing sessions, the number of available trials across treatments for all fish are shown in table 1.

Post-hoc power simulation analysis using the R package, 'simr', assessed the actual observed power of the results produced, given the true effect sizes. A simulation on the generalised linear mixed effects model assessing the effect of the fixed effect of treatment and random effect of fish ID on distance estimates was carried out first with 100 iterations and again with 1000 iterations. The 95% confidence interval for observed power was 96.38-100.00 for 100 iterations and 99.30-100.00 for 1000 iterations. We therefore conclude that the desired power was achieved even under the modified sample size.

1.3 Full distance training protocol

Following a period of pre-training, involving fish learning the association between tweezers and food, and being trained to voluntarily swim into a 2l transport container for a food reward, fish were trained to swim 0.80m to an overhead infrared detector to switch on the aquarium lights and return home for a food reward (main text, figure 1).

Initially, fish were rewarded at the infrared detector concurrently with the light flash and then immediately provided with a food reward in the start area. This was to teach fish that they had to swim out as far as the infrared detector and then return home for a food reward. As they learned the association between the light and food, a reward was then presented only in the start area as the light was switched on. Food was subsequently presented increasingly later during the return journey. By the end of training, the fish had to return to the start area and the door closed before the food reward was provided. Training sessions lasted until 10 correct trials were complete or until 10 minutes had passed, whichever came first. Fish were deemed to have completed training when they swam directly out to the light and returned fully to the start area prior to being presented with and given a food reward on 80% of trials across three consecutive sessions.

Comparison	z-ratio	p-value
Test 1 (0.02m stripes): Test 2 (0.01m stripes)	-18.3	<0.001
Test 1 (0.02m stripes): Test 3 (0.02m check)	-3.08	0.0110
Test 1 (0.02m stripes): Test 4 (horizontal stripes)	5.26	<0.001
Test 2 (0.01m stripes): Test 3 (0.02m check)	14.4	<0.001
Test 2 (0.01m stripes): Test 4 (horizontal stripes)	21.8	<0.001
Test 3 (0.02m check): Test 4 (horizontal stripes)	7.77	<0.001

Supplementary Table 2: Pairwise comparisons of visual treatments, using the Tukey method for comparing a family of four estimates. All pairwise comparisons indicate significant differences.

2 Supplementary Results: additional information

All results were analysed using the program R (The R Project, version 3.6.1) and mixed effects models were constructed using the 'lmer' and 'car' packages.

2.1 Distance estimates across visual treatments

To assess the effect of visual treatment on distance estimate distributions, a generalised linear mixed effects model was fitted by maximum likelihood (Laplace approximation) with a gamma distribution family. Visual test treatment was a fixed effect and fish identity was a random effect. A type II Wald Chisquare test revealed the overall model to be significant (${\chi^2}_3$ = 489, p<0.001). Subsequent Tukey pairwise comparisons identified significant treatment effects (table 2).

Inspection of the mixed effects distance estimate model indicated that the assumptions of testing were met. Selecting a gamma distribution ensured model residuals were normally distributed, ascertained by inspecting a qq-plot and histogram of residuals which revealed a better fit than when a gaussian distribution family was used in the original model.

2.2 Absolute turning position by starting area position across visual treatments

If fish perceived themselves to be estimating distance, then there would be consistent systematic shifts in absolute turning position within the tunnel according to start area position. Data was best modelled with a gamma distribution, and generalised linear mixed effects models were constructed for each visual treatment, assessing the effect of start area position on absolute turning position (Absolute turning positon = Start Area Position (fixed effect) + Fish identity (random effect)). Type II Wald Chisquare tests revealed the overall models to be significant for all visual treatments (Test 1, ${\chi^2}_2$ = 109, p<0.001; Test 2, ${\chi^2}_2$ = 46.1, p<0.001; Test 3, ${\chi^2}_2$ = 81.2, p <0.001; Test 4, ${\chi^2}_2$ = 7.62, p = 0.0221).

However, Tukey pairwise comparisons identified that stepwise significant differences for all three start area positions were only observed for the three treatments where translational spatial frequency information was provided across all tested individuals (Test 1, Test 2 and Test 3), table 3 and fig. 3a-c. Removing translational spatial frequency in test 4 resulted in significant pairwise comparisons only being observed for the comparison between start area positions +0 and +20, and +0 and +10. To be certain that fish are estimating distance, all pairwise treatments need to be significantly different. Inspecting individual fish responses across test treatments can explain the apparent shifting trend in test 4 (fig. 3d). Fish A, B and F exhibit no consistent pairwise shifts according to start area position. For Fish A, the median turning position increases between position +0 and +10, but declines again for position +20. A similar trend is observed for Fish F. Fish B demonstrates no shift between position +0 and +10, and only a very small shift in position +20. The significant trend is therefore likely to be driven by the random effects of Fish D and E. Fish D and E swam to the end of the tunnel on some but not all trials. Trials in which fish swam to the end of the tunnel coincided with the start area in positions +10 and +20 for Fish D and +20 for Fish E. Fish in this visual treatment also exhibited a higher incidence of behavioural stress responses and were less consistent in returning into the start area

between trials, providing further indication of the loss of distance reporting abilities upon the removal of optic flow.

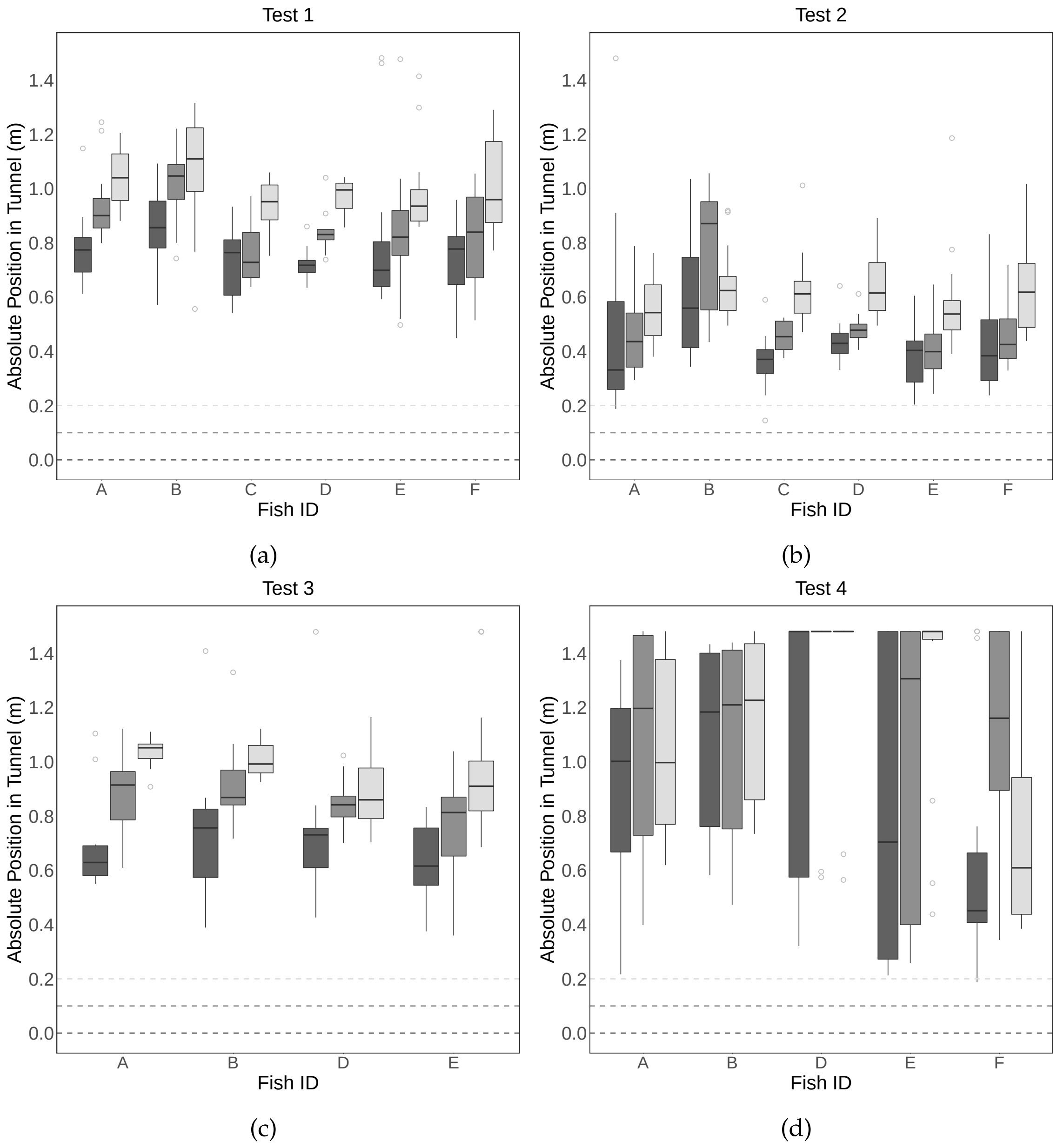

Supplementary Figure 3: Absolute turning positions for individual fish across start area positions. Pairwise shifts are observed across almost all individuals for Tests 1-3 where translational optic flow information is provided (a) - (c). When translational optic flow information was removed (d), there were no consistent shifts in turning position, with a high variability in response across fish identities.

Model	Comparison	z-ratio	p-value
Test 1	+0 : +10	4.56	<**0.001**
Test 1	+10 : +20	5.86	<**0.001**
Test 1	+0 : +20	10.3	<**0.001**
Test 2	+0 : +10	2.95	**0.0089**
Test 2	+10 : +20	3.87	<**0.001**
Test 2	+0 : +20	6.58	<**0.001**
Test 3	+0 : +10	5.66	<**0.001**
Test 3	+10 : +20	3.53	**0.0012**
Test 3	+0 : +20	9.00	<**0.001**
Test 4	+0 : +10	2.50	**0.0337**
Test 4	+10 : +20	-0.034	0.999
Test 4	+0 : +20	2.43	**0.0402**

Supplementary Table 3: Pairwise comparisons of start area position by visual treatments, using the Tukey method for comparing a family of four estimates.

Inspection of the mixed effects models indicate that the assumptions of testing were met. A gamma distribution was selected, as the model residuals were closer to a normal distribution than when fitted with a gaussian distribution. Normality was ascertained by inspecting qq-plots and histograms of the model residuals.

Comparison	z-ratio	p-value
Test 1 (0.02m stripes): Test 2 (0.01m stripes)	1.33	0.546
Test 1 (0.02m stripes): Test 3 (0.02m check)	1.45	0.466
Test 1 (0.02m stripes): Test 4 (horizontal stripes)	11.8	<0.001
Test 2 (0.01m stripes): Test 3 (0.02m check)	0.265	0.994
Test 2 (0.01m stripes): Test 4 (horizontal stripes)	11.0	<0.001
Test 3 (0.02m check): Test 4 (horizontal stripes)	9.35	<0.001

Supplementary Table 4: Pairwise comparisons of absolute distance estimate residuals across visual treatments, using the Tukey method for comparing a family of four estimates.

2.3 Distance estimate variability

Analysing the distance estimate error profiles could be used to investigate the role of visual motion information for odometry. For each distance estimate produced across the four visual treatments, the residuals of estimates from the mean were calculated. These were then converted to absolute error values to permit a standardised comparison of absolute distance estimate variability across treatments. A generalised linear mixed effects model was constructed with a gamma family distribution to test the effect of visual treatment on absolute residuals (fig. 4). Treatment was designated as a fixed effect, and fish identity as a random effect. The overall model was found to be significant (${\chi^2}_3$ = 275, $p<0.001$), and subsequent Tukey pairwise comparisons revealed that only test 4, where fish had access to no spatial frequency information, was different from the other four treatments (table 4). This indicates that access to spatial frequency information is necessary for accurate distance estimation.

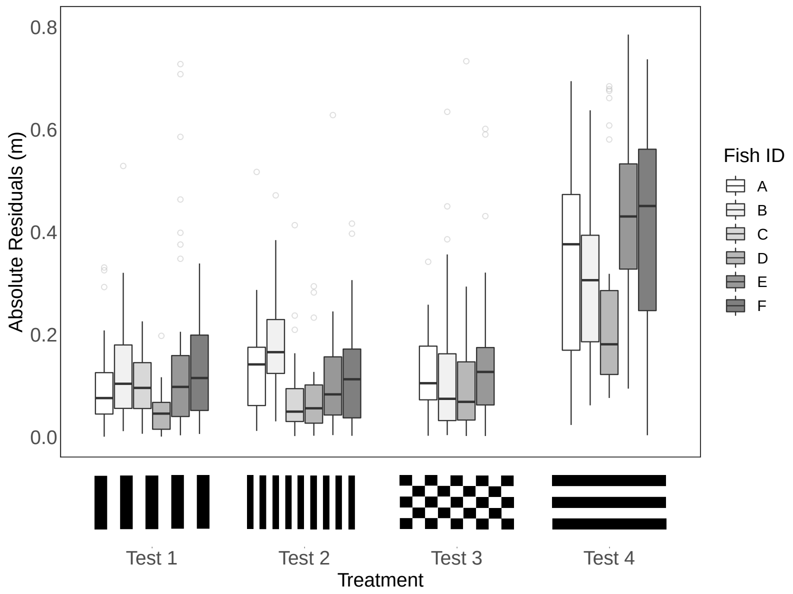

Supplementary Figure 4: Absolute distance estimate residuals from treatment average. Visual treatment was a significant predictor of distance estimate error, but Tukey pairwise comparisons identified that only test 4 with no spatial frequency information provided produced an absolute distance estimate error profile that was significantly different from the other test treatments (table 4). This indicates that fish rely on spatial frequency information for odometry.

2.4 Swimming speed across visual treatments

Inspecting the overall distribution for swimming speeds indicated the data to be strongly right skewed, and a gamma distribution was therefore chosen for all modelling, as it can be used for skewed continuous positive data. A generalised linear mixed effects model was selected to model swimming speed using visual treatment as a fixed effect, and fish identity as a random effect. A type II Wald Chisquare test indicated that swimming speed was found to vary with visual treatment (${\chi^2}_3$ = 102, p <0.001). Subsequent Tukey pairwise comparisons identified that swimming speeds showed small variations with spatial frequency and pattern, with high variability in responses across individuals (table 5 and fig. 5). Mean swimming speeds increased uniformly in test 4 when optic flow information was removed.

To assess the effect of visual test treatment on the variance in swimming speeds, a

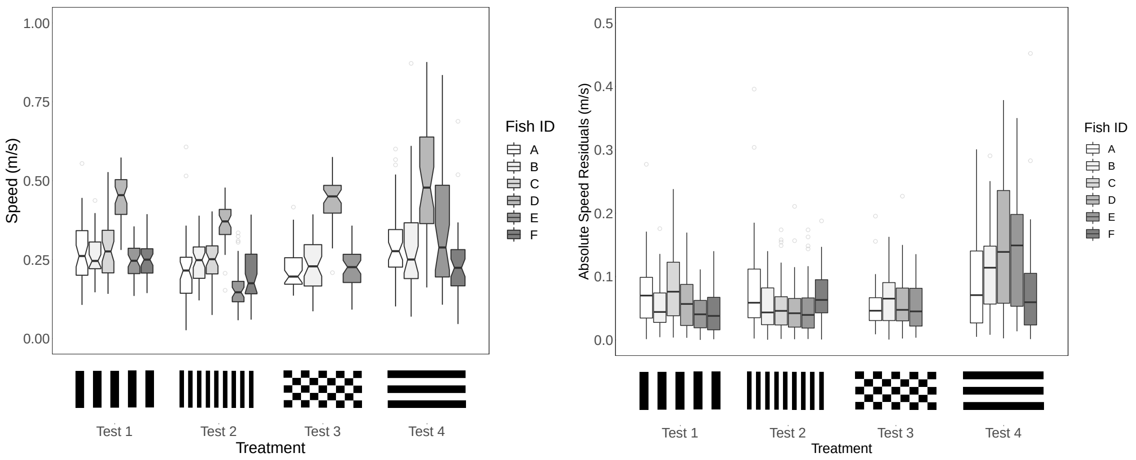

(a) (b)

Supplementary Figure 5: (a) Individual swimming speeds split by treatment and fish identity. Average swimming speeds show slight sensitivity to translational spatial frequency, an effect exhibited more strongly by some individuals than others. (b) Absolute swimming speed residuals split by treatment and fish identity. Swimming speed and absolute residuals of swimming speed (a measure of swimming speed variability) increased uniformly for all fish in test 4.

mixed effects model with visual treatment as a fixed effect and fish identity as a random effect was constructed for the residuals of swimming speeds from the treatment means. A type II Wald Chisquare test indicated that the magnitude of swimming speed residuals varied with visual treatment (${\chi^2}_3$ = 137, p <0.001). Subsequent Tukey pairwise comparisons identified that residuals only increased in test 4 for all tested individuals, where translational optic flow information was removed (table 6).

Inspection of the mixed effects swimming speed and swimming speed residuals models indicated that the assumptions of testing were met. Selecting a gamma distribution ensured model residuals were normally distributed, ascertained by inspecting a qq-plot and histogram of residuals which revealed a better fit than when a gaussian distribution family was used in the original model.

Comparison	z-ratio	p-value
Test 1 (0.02m stripes): Test 2 (0.01m stripes)	-6.42	<0.001
Test 1 (0.02m stripes): Test 3 (0.02m check)	-2.98	0.0154
Test 1 (0.02m stripes): Test 4 (horizontal stripes)	3.58	0.002
Test 2 (0.01m stripes): Test 3 (0.02m check)	2.78	0.0277
Test 2 (0.01m stripes): Test 4 (horizontal stripes)	9.51	<0.001
Test 3 (0.02m check): Test 4 (horizontal stripes)	6.16	<0.001

Supplementary Table 5: Pairwise comparisons of swimming speed across visual treatments, using the Tukey method for comparing a family of four estimates. All pairwise comparisons indicate significant differences.

Comparison	z-ratio	p-value
Test 1 (0.02m stripes): Test 2 (0.01m stripes)	0.189	0.998
Test 1 (0.02m stripes): Test 3 (0.02m check)	-0.342	0.986
Test 1 (0.02m stripes): Test 4 (horizontal stripes)	8.49	<0.001
Test 2 (0.01m stripes): Test 3 (0.02m check)	-0.507	0.958
Test 2 (0.01m stripes): Test 4 (horizontal stripes)	8.32	<0.001
Test 3 (0.02m check): Test 4 (horizontal stripes)	7.50	<0.001

Supplementary Table 6: Pairwise comparisons of swimming speed residuals across visual treatments, using the Tukey method for comparing a family of four estimates. Pairwise comparisons between test 4 and all other treatments indicate significant differences.

References

[1] Cheney, K. L., Newport, C., McClure, E. C. & Marshall, N. J. Colour vision and response bias in a coral reef fish. *The Journal of experimental biology* **216**, 2967–73 (2013).

[2] Newport, C. *et al.* Fish use colour to learn compound visual signals. *Animal Behaviour* **125**, 93–100 (2017).

[3] Cohen, J. *The Concepts of Power Analysis BT - Statistical Power Analysis for the Behavioral Sciences (Revised Edition)* (Hillsdale, N.J. : L. Erlbaum Associates, 1988).

[4] Karlsson, C., Willis, J., Patel, M. & Burt de Perera, T. Teleost fish can accurately estimate distance travelled. *bioRxiv* 834341 (2019).
