# Supplementary material for: Visual odometry of Rhinecanthus aculeatus depends on the visual density of the environment
Source: Commun Biol. 2022 Oct 1;5:1045. doi: 10.1038/s42003-022-03925-5 (PMC9526725; doi:10.1038/s42003-022-03925-5)
Supplement: Supplementary file 3 — Description of Additional Supplementary Files [file 42003_2022_3925_MOESM3_ESM.pdf]

Description of Additional Supplementary Files

File name: Supplementary Data 1:

Description: Raw data file of fish distance estimates prior to calculations to convert pixels into distance estimates in metres, and calculation of movement speed.

File name: Supplementary Data 2:

Description: Distance estimates and movement speeds calculated from the raw pixel data.

File name: Supplementary Data 3:

Description: Calculations performed on the raw pixel distance estimation data to obtain distance estimates in metres, and movement speeds for each trial.
