# Supplementary material for: Visual odometry of Rhinecanthus aculeatus depends on the visual density of the environment
Source: Commun Biol. 2022 Oct 1;5:1045. doi: 10.1038/s42003-022-03925-5 (PMC9526725; doi:10.1038/s42003-022-03925-5)
Supplement: Supplementary file 7 — Reporting Summary [file 42003_2022_3925_MOESM7_ESM.pdf]

nature research

Corresponding author(s): DBPR COMMSBIO-20-0998-T

Last updated by author(s): Sep 25, 2020

Reporting Summary

Nature Research wishes to improve the reproducibility of the work that we publish. This form provides structure for consistency and transparency in reporting. For further information on Nature Research policies, see Authors & Referees and the Editorial Policy Checklist.

Statistics

For all statistical analyses, confirm that the following items are present in the figure legend, table legend, main text, or Methods section.

n/a	Confirmed	
☐	☒	The exact sample size (*n*) for each experimental group/condition, given as a discrete number and unit of measurement
☐	☒	A statement on whether measurements were taken from distinct samples or whether the same sample was measured repeatedly
☐	☒	The statistical test(s) used AND whether they are one- or two-sided<br>*Only common tests should be described solely by name; describe more complex techniques in the Methods section.*
☐	☒	A description of all covariates tested
☐	☒	A description of any assumptions or corrections, such as tests of normality and adjustment for multiple comparisons
☐	☒	A full description of the statistical parameters including central tendency (e.g. means) or other basic estimates (e.g. regression coefficient) AND variation (e.g. standard deviation) or associated estimates of uncertainty (e.g. confidence intervals)
☐	☒	For null hypothesis testing, the test statistic (e.g. *F*, *t*, *r*) with confidence intervals, effect sizes, degrees of freedom and *P* value noted<br>*Give P values as exact values whenever suitable.*
☒	☐	For Bayesian analysis, information on the choice of priors and Markov chain Monte Carlo settings
☐	☒	For hierarchical and complex designs, identification of the appropriate level for tests and full reporting of outcomes
☐	☒	Estimates of effect sizes (e.g. Cohen's *d*, Pearson's *r*), indicating how they were calculated

Our web collection on statistics for biologists contains articles on many of the points above.

Software and code

Policy information about availability of computer code

Data collection	Video data was collected using a Point Grey USB3 camera controlled using StreamPix 7 Video Capture Software.
Data analysis	Behavioural distance estimation data was extracted as pixel coordinates from jpeg frames of the videos using software written in MatLab (Mathworks Inc.).<br>Statistical Analyses and graphing were conducted in 'R' version 3.6.1

For manuscripts utilizing custom algorithms or software that are central to the research but not yet described in published literature, software must be made available to editors/reviewers. We strongly encourage code deposition in a community repository (e.g. GitHub). See the Nature Research guidelines for submitting code & software for further information.

Data

Policy information about availability of data

All manuscripts must include a data availability statement. This statement should provide the following information, where applicable:

- Accession codes, unique identifiers, or web links for publicly available datasets
- A list of figures that have associated raw data
- A description of any restrictions on data availability

The data that support the findings of this study, and associated R code for processing the data are available from the corresponding author upon request.

Field-specific reporting

Please select the one below that is the best fit for your research. If you are not sure, read the appropriate sections before making your selection.

☐ Life sciences ☒ Behavioural & social sciences ☐ Ecological, evolutionary & environmental sciences

For a reference copy of the document with all sections, see nature.com/documents/nr-reporting-summary-flat.pdf

Behavioural & social sciences study design

All studies must disclose on these points even when the disclosure is negative.

Study description

We tested whether the marine fish, Rhinecanthus acuelatus, uses visual motion information to estimate travel distances. Fish were trained to a sample distance, and tested in their ability to report the previously learned distance following a series of manipulations to the visual background. All distance estimates are quantitative results.

Research sample

Subjects were 6 adult wild-caught Rhinecanthus aculeatus specimens from the Maldives, sourced through a local supplier. While it is not possible to determine sex or age of live Rhinecanthus specimens, all tested individuals were of standard length (nose to the caudal peduncle) 10-14cm.

Sampling strategy

As individuals were wild-caught and could not be returned to the wild following experiments, we sought to minimise the number of individuals used whilst achieving appropriate statistical power. The required sample size was determined in 'R' using a-priori power analysis, assuming a large effect size in a four-way ANOVA comparisson, using a Cohen's d approximation of 0.8. This indicated that 5.3 individuals were required to achieve appropriate power. Further post-hoc simulations on observed power of our data was performed using the package 'simr' in R.

Data collection

All behavioural training and data collection was conducted by the corresponding author. Testing sessions were filmed from above with a Point Grey USB3 camera controlled using StreamPix 7 video capture software. Qualifying distance estimate trials, as outlined in the methods of the main manuscript, were subsequently converted to jpegs and the turning position of each distance estimate trial was extracted as an x,y pixel coordinate using a MatLab manual video tracking program. Pixel coordinates were converted to distance coordinates using the conversion: 0.01m = 14.4 pixels.

Timing

All individuals arrived in the lab from the wild and subsequently reached testing criterion at different stages, owing to different learning rates between individuals. All data collection took place between 01/12/2018 and 18/10/2019. No individual had been laboratory housed for more than 9 months prior to experiment completion.

Data exclusions

Testing session 9 of Test 3 (checkerboard treatment) for Fish B (lab name Degas) was excluded from analysis because the testing session was disturbed by a fire alarm and entry of additional people not present during training or any other testing sessions into the testing room. The fire alarm was a false alarm, and the testing session was completed to avoid undue stress to the fish by removing him from the experiment arena shortly after moving him out of his home tank originally. This session was excluded and repeated as it could not be ascertained to what extent the fish became distracted from the distance estimation task as a result of the fire alarm itself or additional people entering the lab.
Fish E, test 1 session 6: trial 5 was excluded due to experimenter straightening experiment apparatus during distance estimate. Sessions 4 and 7 were excluded due to stress upon transfer via the transport container into the experiment tank, and subsequent visible stress behaviours during testing sessions. These sessions were repeated in two sessions achieving 5 trials with no visible stress responses.
All other distance estimate trials were included according to the criteria outlined in the methods section. If sampling exceeded the required 45 trials per treatment (of which 15 were from each start area position), distance estimates were preferentially extracted from sessions achieving the greater number of successful trials.

Non-participation

All six individuals completed distance training and the baseline test treatment (treatment 1). Four fish (ID: A, B, D and E) completed all four testing treatments, but Fish C and F experienced loss of motivation to continue the distance estimation task following Test 2 and Test 3 respectively. These fish were unable to reliably complete the 5 training trials at 80% accuracy that preceded each block of 5 testing trials. When this continued across over 10 consecutive sessions, fish were removed from experiments. Sample sizes across treatments were therefore as follows:
Treatment 1 (0.02m stripes, baseline): 6 fish
Treatment 2 (0.01m stripes): 6 fish
Treatment 3 (0.02m checkerboard): 4 fish
Treatment 4 (horizontal stripes): 5 fish

Randomization

All participants were allocated to complete all experimental treatments at the start of experiments. Deviations from this were a result of participants being removed from experiments (see above note on non-participation).

Reporting for specific materials, systems and methods

We require information from authors about some types of materials, experimental systems and methods used in many studies. Here, indicate whether each material, system or method listed is relevant to your study. If you are not sure if a list item applies to your research, read the appropriate section before selecting a response.

Materials & experimental systems

n/a	Involved in the study	
☒	☐	Antibodies
☒	☐	Eukaryotic cell lines
☒	☐	Palaeontology
☐	☒	Animals and other organisms
☒	☐	Human research participants
☒	☐	Clinical data

Methods

n/a	Involved in the study	
☒	☐	ChIP-seq
☒	☐	Flow cytometry
☒	☐	MRI-based neuroimaging

Animals and other organisms

Policy information about studies involving animals; ARRIVE guidelines recommended for reporting animal research

Laboratory animals	The study involved wild-caught subjects that were subsequently housed in the laboratory for the duration of experiments. All subjects were required to be between 10-14cm standard length.
Wild animals	Wild Rhinecanthus aculeatus subjects were sourced from the wild on reefs in the Maldives through a local supplier based in Oxford (The Goldfish Bowl). Subjects were caught and transported into the UK in accordance with regulations, and delivered directly to the laboratory in Oxford. Subjects went through a period of quarantine and monitored for any illness and stress responses prior to inclusion in the main aquarium system. Following experiments, individuals were re-homed to the local supplier (Goldfish Bowl) to be sold on as ornamental aquarium fish in the pet trade, or used in school outreach schemes on coral reef environments.
Field-collected samples	There were no samples collected from the field.
Ethics oversight	This work did not fall under any regulated procedures outlined by ASPA, but was approved by a local ethics committee prior to starting experiments (AWERB – Animal Welfare Ethical Review Body, University of Oxford. Project code: APA/1/5/ZOO/NASPA/Burt/PathIntegration).

Note that full information on the approval of the study protocol must also be provided in the manuscript.
